# Associations between perceived campus walkability and self-reported exercise behavior among college students: a cross-sectional serial indirect-effects study of exercise motivation and general self-efficacy

**DOI:** 10.3389/fpubh.2026.1871624

**Published:** 2026-09-04

**Authors:** Guanghui Liu, Jianmin Zhao, Mingyu Zhao, Yang Zhao, Congcong Zhao

**Affiliations:** 1 School of Sports and Leisure, Shandong Sport University, Jinan, Shandong, China; 2 School of Humanities, Beijing Sport University, Beijing, China; 3 School of Politics and Public Administration, Qufu Normal University, Rizhao, Shandong, China; 4 Foreign Language College, Weifang University, Weifang, Shandong, China; 5 School of Basic-Level Two Committees Education, Shandong Open University, Jinan, China

**Keywords:** college students, cross-sectional indirect association, exercise behavior, exercise motivation, general self-efficacy, perceived campus walkability

## Abstract

**Objective:**

This study examined the cross-sectional association between perceived campus walkability and self-reported exercise behavior among college students and explored indirect associations through exercise motivation and general self-efficacy under a prespecified serial ordering. The study aimed to inform the development of health-promoting university campuses and strategies to support student exercise behavior.

**Methods:**

A cross-sectional online questionnaire survey was conducted among 1,739 undergraduate students recruited through multi-institutional convenience sampling from six undergraduate universities located in different regions of Shandong Province, China. Perceived campus walkability, exercise motivation, general self-efficacy, and exercise behavior were assessed using self-report measures. Descriptive statistics, Pearson correlations, and hierarchical multiple linear regression with demographic and background covariates were used to examine associations. Heteroscedasticity-consistent HC3 standard errors were reported. Cross-sectional indirect associations under the prespecified ordering of perceived campus walkability → exercise motivation → general self-efficacy → exercise behavior were estimated using PROCESS version 4.2 Model 6 with 5,000 bootstrap resamples. Because all variables were measured at one time point, the indirect-effect analysis was not interpreted as establishing temporal mediation or causality.

**Results:**

The mean scores were 136.99 (SD 15.86) for perceived campus walkability, 58.00 (SD 11.68) for exercise motivation, 26.90 (SD 7.11) for general self-efficacy, and 16.81 (SD 19.88) for exercise behavior. Exercise intensity was classified as light for 714 participants (41.1%), low for 400 (23.0%), moderate for 264 (15.2%), vigorous but non-sustained for 303 (17.4%), and vigorous and sustained for 58 (3.3%). Perceived campus walkability was positively correlated with exercise motivation (*r* = 0.237), general self-efficacy (*r* = 0.215), and exercise behavior (*r* = 0.127). Exercise motivation (*r* = 0.142) and general self-efficacy (*r* = 0.170) were also positively correlated with exercise behavior (all *p* < 0.001). In the fully adjusted model, perceived campus walkability (*B* = 0.066, HC3 SE = 0.028, 95% CI 0.011 to 0.121, *p* = 0.019) and exercise motivation (*B* = 0.141, HC3 SE = 0.044, 95% CI 0.055 to 0.227, *p* = 0.001) were positively associated with exercise behavior. The coefficient for general self-efficacy was not statistically distinguishable from zero (*B* = 0.141, HC3 SE = 0.076, 95% CI − 0.009 to 0.291, *p* = 0.065). The indirect association through exercise motivation was 0.0243 (95% bootstrap CI 0.0096 to 0.0413). In contrast, the indirect association through general self-efficacy (0.0051, 95% bootstrap CI − 0.0004 to 0.0123) and the serial indirect association through exercise motivation and general self-efficacy (0.0076, 95% bootstrap CI −0.0007 to 0.0159) included zero. Competing and reverse-direction models also yielded nonzero indirect associations, indicating that the assumed temporal ordering could not be determined from these cross-sectional data.

**Conclusion:**

Perceived campus walkability showed a small positive association with self-reported exercise behavior among college students. Exercise motivation accounted for a statistically nonzero indirect association under the prespecified model, whereas indirect associations through general self-efficacy alone and through the serial pathway were not supported. These findings support combining improvements in campus destinations and walking access with strategies that strengthen students’ motivation to exercise; however, longitudinal or experimental studies are needed to determine temporal direction and causal mechanisms.

## 1 Introduction

Insufficient physical activity is a major global public health concern. Physical activity includes bodily movement undertaken in daily living, transportation, work, recreation, and structured exercise (1). Regular participation is associated with lower risks of cardiovascular disease, type 2 diabetes, some cancers, premature mortality, and adverse mental health outcomes (2, 3). Nevertheless, approximately 31% of adults worldwide did not meet recommended physical activity levels in 2022, and the prevalence of insufficient activity has increased since 2010 (2, 4). These trends underscore the importance of identifying environmental and psychological factors associated with active behavior in populations undergoing major life transitions.

College students constitute one such population. The transition to university is often accompanied by increased academic demands, prolonged sedentary time, greater screen use, irregular routines, and reduced participation in organized physical education. The World Health Organization recommends that adults accumulate 150–300 min of moderate-intensity aerobic physical activity per week, 75–150 min of vigorous-intensity activity, or an equivalent combination, while also limiting sedentary time (5). However, students’ activity opportunities are shaped not only by individual preferences but also by the environments in which they study, live, travel, and socialize. The university campus is therefore an important setting for examining how environmental perceptions and individual psychological characteristics are related to exercise behavior.

Walkability refers to the extent to which an environment supports walking through characteristics such as destination accessibility, land-use mix, street connectivity, pedestrian infrastructure, aesthetics, and traffic safety (6). In a university setting, distances between residences, teaching buildings, libraries, dining facilities, service destinations, and sports facilities may influence how students navigate the campus. Pedestrian routes, lighting, traffic conditions, greenery, and perceived safety may also shape students’ experiences of campus movement. Importantly, perceived walkability is not equivalent to objectively measured walkability. Students’ evaluations may be influenced by familiarity with the campus, prior use of its spaces, mobility preferences, affective states, and existing activity levels. Accordingly, the present study concerns students’ perceptions of campus walkability rather than directly measured features of campus design.

Previous studies have reported associations between campus environments and students’ walking-related outcomes. Liao et al. (7) found that perceived campus walkability was related to affective walking experiences and that walking attitudes showed an indirect association with these experiences. Wang et al. (8), using the Neighborhood Environment Walkability Scale and the International Physical Activity Questionnaire among students from two Chinese universities, reported associations of land-use diversity, accessibility, and infrastructure with walking behavior. Zhang et al. (9) combined questionnaires, field audits, and geographic information system data across eight universities and found that several campus design and street-level characteristics were associated with pedestrian volume. Together, this evidence suggests that campus environments may be relevant to walking and spatial use. However, perceived walkability, objective campus characteristics, daily walking, and deliberate exercise are distinct constructs and should not be treated as interchangeable.

The outcome assessed in the present study was the Physical Activity Rating Scale-3 (PARS-3), which combines reported exercise intensity, duration, and frequency. PARS-3 primarily reflects deliberate exercise behavior and does not comprehensively measure active commuting, incidental campus walking, household activity, or total movement. Therefore, the present study uses the term self-reported exercise behavior rather than total physical activity. Although perceived campus walkability may be more directly related to walking and active travel, it may also be associated with deliberate exercise if students who perceive their campus as accessible, safe, and pleasant experience fewer environmental barriers to being active. The extent of this association, however, is uncertain and may be small because exercise behavior is influenced by many demographic, academic, social, and psychological factors.

Exercise motivation is one psychological characteristic that may be associated with both perceived environmental support and exercise behavior. The exercise motivation measure used in this study assesses motives related to health, weight and appearance, enjoyment, skill development, and social interaction. These motive domains describe reasons for engaging in exercise; they do not directly measure the autonomous and controlled behavioral regulations specified in self-determination theory. Nevertheless, broader self-determination research indicates that motivational quality is relevant to sustained exercise participation (10). A campus perceived as convenient and supportive may be associated with more favorable exercise motives, although the cross-sectional design of the present study cannot establish whether environmental perceptions precede motivation.

The study also assessed general self-efficacy, defined as a broad belief in one’s capacity to manage difficult or unexpected situations. General self-efficacy differs from exercise-specific or barrier-specific self-efficacy and should not be interpreted as direct confidence in initiating or maintaining exercise. It may nevertheless be related to exercise behavior because individuals with stronger general coping beliefs may be more persistent in pursuing personally valued activities. Exercise motivation and general self-efficacy may also be related to each other. One possible ordering is that stronger exercise motives coincide with successful engagement and competence experiences, which are subsequently associated with broader efficacy beliefs. Zhao et al. (11) reported relationships among exercise motivation, exercise climate, exercise-specific self-efficacy, and exercise behavior in college students. However, because the present study measured general rather than exercise-specific self-efficacy, this evidence provides only indirect theoretical support for the proposed ordering.

Alternative temporal orderings are equally plausible. General self-efficacy may precede exercise motivation, more active students may report stronger motivation and efficacy because of successful exercise experiences, and students who use campus spaces more frequently may evaluate campus walkability more favorably. Shared determinants—including sports-related study, health consciousness, perceived fitness, social support, and socioeconomic conditions—may also contribute to the observed associations. A cross-sectional serial indirect-effects model can quantify indirect associations under an assumed ordering of variables, but it cannot establish temporal precedence, causality, or a psychological mechanism. Longitudinal data collected across at least three measurement occasions would be required to evaluate the proposed temporal sequence more appropriately.

Against this background, the present cross-sectional study examined associations among perceived campus walkability, exercise motivation, general self-efficacy, and self-reported exercise behavior in 1,739 undergraduate students. The primary analysis estimated indirect associations under the prespecified ordering of perceived campus walkability → exercise motivation → general self-efficacy → exercise behavior. To evaluate the uncertainty of this ordering, a competing model placing general self-efficacy before exercise motivation and a reverse-direction model were also examined. The study was intended to provide exploratory evidence concerning environmental perceptions and psychological correlates of exercise behavior, rather than to confirm a causal mediation mechanism or the effects of modifying campus infrastructure.

The following hypotheses were specified:

*H1*: More favorable perceived campus walkability is positively associated with self-reported exercise behavior after adjustment for relevant demographic and background characteristics.

*H2*: Exercise motivation shows a positive indirect association between perceived campus walkability and self-reported exercise behavior under the specified cross-sectional model.

*H3*: General self-efficacy shows a positive indirect association between perceived campus walkability and self-reported exercise behavior under the specified cross-sectional model.

*H4*: A positive serial indirect association is observed under the prespecified ordering of perceived campus walkability → exercise motivation → general self-efficacy → self-reported exercise behavior. Because temporal order was not observed, this hypothesis is evaluated alongside theoretically plausible competing and reverse-direction models.

## 2 Participants and methods

### 2.1 Study design, recruitment, and participants

This study used a cross-sectional online questionnaire design. Participants were recruited through multi-institutional convenience sampling from six degree-granting undergraduate universities located in different regions of Shandong Province, China. Volunteer recruiters at each participating university assisted in distributing the online questionnaire link or QR code to eligible undergraduate students. Data were collected from June 29 to July 7, 2024, and the survey platform exported 2,255 response records. The eligibility criteria were undergraduate status, age 18–29 years, and a correct response to the embedded attention-check item (“strongly agree”). Before examining associations among the study variables, ineligible records were excluded sequentially according to the prespecified criteria. First, 16 records from respondents outside the eligible age range were excluded, followed by 80 records from postgraduate students. Among the remaining undergraduate records, 420 failed the attention check and were excluded. The final analytic sample therefore comprised 1,739 students. Responses to the principal study variables were complete in the exported dataset; consequently, no participants were excluded because of item-level missingness, and no missing-data imputation was performed.

Among the 1,739 students included in the analysis, 889 (51.1%) were male and 850 (48.9%) were female, with a mean age of 20.60 years (SD 1.56). The sample comprised 812 first-year, 385 s-year, 353 third-year, and 189 fourth-year students. Academic disciplines included arts or sports-related disciplines (*n* = 370), humanities and social sciences (*n* = 427), science-related disciplines (*n* = 599), and engineering-related disciplines (*n* = 343). A total of 1,010 students (58.1%) identified as Han Chinese, and 729 (41.9%) identified as members of ethnic minority groups.

Participation was voluntary, and data were collected anonymously. Before completing the questionnaire, participants were informed of the study purpose, survey content, confidentiality safeguards, voluntary nature of participation, and their right to decline or withdraw from the study. All participants provided electronic informed consent. The analytic dataset contained no directly identifying information. The study was approved by the Sports Science Ethics Committee of Shandong Sport University (Approval No. 2025089), before participant recruitment and data collection commenced, and was conducted in accordance with the Declaration of Helsinki.

The online survey platform required participants to log in before responding and was configured to permit only one submission from each authenticated account, device, and IP address. These restrictions were implemented to reduce the likelihood of duplicate participation. The online survey platform required participants to log in before responding and was configured to permit only one submission from each authenticated account, device, and IP address. These restrictions were implemented to reduce the likelihood of duplicate participation. However, because account, device, and IP-address identifiers were not retained in the de-identified analytic dataset, compliance with these duplicate-submission controls could not be independently verified.

Although participants were recruited from six undergraduate universities, the de-identified analytic dataset did not retain individual-level identifiers for university, campus, or class membership. Participants could therefore not be reassigned to their original university-, campus-, or class-level clusters. Without making unsupported assumptions, it was not possible to calculate cluster-robust standard errors or estimate multilevel models at these levels. The statistical analyses therefore treated individual observations as independent. Unaccounted within-university, within-campus, or within-class correlations may have resulted in underestimated standard errors and overly narrow confidence intervals. This issue is acknowledged as a study limitation.

No *a priori* sample-size calculation was conducted or documented before data collection. Accordingly, we did not report a *post hoc* power analysis based on the observed effects. The precision of the estimates was evaluated primarily using 95% confidence intervals, and the absence of an *a priori* sample-size calculation is acknowledged as an additional limitation.

### 2.2 Measures

The questionnaire included sociodemographic characteristics, perceived campus walkability, self-reported exercise behavior, exercise motivation, and general self-efficacy. The complete item wording and response options are provided in Appendices 1–4; scoring and analytic coding are summarized in Appendix 5.

#### 2.2.1 Sociodemographic characteristics

The survey recorded sex, age, ethnicity, family residence, average monthly living expenses, grade, academic discipline, campus residence, height, weight, perceived body shape, parental educational attainment, and perceived family economic status. Covariates were selected *a priori* on substantive grounds because demographic, academic, residential, and socioeconomic characteristics could plausibly be associated with both environmental perceptions and exercise behavior. Nominal variables with more than two categories were represented by indicator variables with prespecified reference categories; they were not entered as single continuous numeric codes. Ordinal variables were analyzed categorically in the primary models, with trend coding examined only as a sensitivity analysis. The same adjustment set was used in the relevant mediator and outcome equations unless explicitly stated otherwise.

#### 2.2.2 Perceived campus walkability

Perceived campus walkability was assessed using a campus-adapted questionnaire informed by the Neighborhood Environment Walkability Scale (NEWS) (6). NEWS assesses perceived destination accessibility, street connectivity, pedestrian infrastructure, aesthetics, and traffic safety. The present instrument used campus-specific destinations and wording; it should therefore be regarded as an adapted perceived-environment measure rather than the original NEWS or NEWS-A.

The source questionnaire contained 42 substantive items: 13 destination walking-time items and 29 agreement items. However, the analytic export contained 13 walking-time items and 28 agreement items; the item concerning pedestrian density during peak periods was not present. Accordingly, the analyses were based on the 41 substantive walkability items available in the dataset, and the omitted item was not imputed. Walking-time responses were coded so that shorter times represented better accessibility (1–5 min = 5, 6–10 min = 4, 11–20 min = 3, 20–30 min = 2, >30 min = 1). “Do not know” was coded 0 for that destination and was not treated as an item-level missing value. Agreement items were scored from 1 (strongly disagree) to 5 (strongly agree), with negatively worded items reverse-coded before scale construction. Item-level wording, coding direction, and the discrepancy between the questionnaire and analytic export are shown in Appendices 2, 5.

Because the instrument combines different response formats and represents several environmental domains, internal consistency of a single total score is not sufficient evidence of structural validity. Primary analyses therefore used the prespecified composite score, with sensitivity analyses conducted for the available walkability domains. The adaptation process and the absence of a complete validation study—including confirmatory factor analysis, convergent validity, and discriminant validity—are treated as measurement limitations rather than resolved by Cronbach’s alpha alone.

#### 2.2.3 Self-reported exercise behavior

Exercise behavior was measured using the Physical Activity Rating Scale-3 (PARS-3), which contains one item each for exercise intensity, duration, and frequency (12). Intensity and frequency were coded 1–5. Duration was coded 0–4 for <10, 11–20, 21–30, 31–59, and ≥60 min, respectively. The composite score was calculated as intensity × duration × frequency and ranged theoretically from 0 to 100, with higher scores indicating a higher self-reported exercise level. The complete item wording and response categories are provided in Appendix 1.

PARS-3 primarily assesses deliberate exercise and does not comprehensively capture active commuting, incidental campus walking, household activity, daily steps, or total movement. The outcome is therefore termed self-reported exercise behavior throughout the manuscript. The intensity labels are response-category descriptions, not separate diagnostic classifications. Because intensity, duration, and frequency form a multiplicative activity index rather than interchangeable reflective indicators of a single latent construct, Cronbach’s alpha was not used as evidence of reliability for PARS-3. Criterion validity against accelerometry or pedometer data was not evaluated in the present sample.

#### 2.2.4 Exercise motivation

Exercise motivation was assessed with the 15-item abbreviated exercise motivation questionnaire supplied with the study. It contains five three-item domains: health, weight and appearance, enjoyment, competence or skill development, and social interaction. Each item was rated from 1 (completely inconsistent) to 5 (completely consistent); no item was reverse-scored. Domain scores ranged from 3 to 15 and the summed score ranged from 15 to 75, with higher values indicating stronger endorsement of the included exercise motives. Complete items are provided in Appendix 3.

These domains describe reasons for exercise but do not map directly onto the autonomous motivation, controlled motivation, and amotivation regulations of self-determination theory. Consequently, the study does not claim to test a self-determination theory measurement model. Domain-level descriptive statistics and reliability estimates are reported in addition to the total score. The use of the total score is treated as a pragmatic composite analysis and is interpreted cautiously pending confirmation of the instrument’s factor structure.

#### 2.2.5 General self-efficacy

General self-efficacy was measured using the 10-item Chinese version of the General Self-Efficacy Scale developed by Schwarzer and Jerusalem (13). Items were rated from 1 (completely incorrect) to 4 (completely correct), with no reverse-scored items. Item scores were summed to produce a total ranging from 10 to 40; higher scores indicated stronger general beliefs about coping with difficult or unexpected situations. Complete items are provided in Appendix 4.

The instrument measures general rather than exercise-specific or barrier-specific self-efficacy. It was therefore labeled general self-efficacy in all analyses and was not interpreted as direct confidence in initiating or maintaining exercise. Internal consistency was evaluated using both Cronbach’s alpha and McDonald’s omega, while recognizing that reliability coefficients do not establish construct validity.

### 2.3 Data quality and missing data

The questionnaire used neutral instructions, anonymous responses, varied response formats, and embedded attention checks. The principal attention item instructed respondents to select “strongly agree.” Eligibility and quality-control exclusions were defined before association analyses. The final analytic dataset had no item-level missing values for the principal measures; therefore, complete-case deletion and multiple imputation were not required for those variables.

Harman’s single-factor analysis was retained only as a descriptive diagnostic. The first unrotated factor explained 23.132% of the total variance and no dominant single factor emerged. This result does not demonstrate the absence of common method variance. All principal constructs were self-reported by the same respondents at the same time, so shared response style, social desirability, affect, and evaluation tendencies remain plausible sources of bias.

### 2.4 Statistical analysis

Analyses were conducted using IBM SPSS Statistics 27.0 and PROCESS version 4.2 (14). Categorical variables were summarized using counts and percentages, and continuous variables using means, standard deviations, medians, and interquartile ranges. Distributional characteristics of the PARS-3 score were examined using histograms, Q–Q plots, skewness, and the frequency of zero scores. Group comparisons report sample sizes, means, standard deviations, test statistics, degrees of freedom, exact *p* values, and effect sizes; multiplicity-adjusted *post hoc* comparisons were used when an omnibus test involved more than two groups.

Associations among the principal variables were summarized using Pearson correlations, with Spearman correlations used as a sensitivity analysis when distributions were markedly non-normal or influential observations were evident. Before linear modeling, linearity, residual distribution, homoscedasticity, multicollinearity, leverage, influential observations, and model specification were assessed. Variance inflation factors and tolerance values were reported. Heteroscedasticity-consistent HC3 standard errors and bootstrap confidence intervals were used as sensitivity analyses when conventional homoscedasticity assumptions were not supported.

Hierarchical multiple regression was used to distinguish the contribution of the focal variables from that of background characteristics. Model 1 contained the prespecified demographic and background covariates. Model 2 added perceived campus walkability. Model 3 added exercise motivation and general self-efficacy. For each step, unstandardized B, standard error, standardized β, 95% confidence interval, *p* value, adjusted R^2^, change in R^2^, and change in F were reported. All nominal covariates were dummy-coded, and reference categories were identified in the relevant table note.

Cross-sectional indirect associations were estimated with PROCESS Model 6 using 5,000 bootstrap resamples and 95% bootstrap confidence intervals. The prespecified model ordered perceived campus walkability → exercise motivation → general self-efficacy → self-reported exercise behavior. This ordering was theoretical and was not observed temporally. The analysis therefore estimated direct and indirect associations under an assumed ordering; it did not establish mediation, temporal precedence, or causality. An indirect association was considered statistically distinguishable from zero when its bootstrap confidence interval excluded zero.

Two alternative specifications were examined to assess ordering uncertainty: a competing model placing general self-efficacy before exercise motivation, and a reverse-direction model in which self-reported exercise behavior preceded the psychological variables. The estimates from these models were interpreted as comparative cross-sectional associations, not tests that identify the true temporal sequence. Longitudinal data across at least three measurement occasions would be required to evaluate the proposed sequence more appropriately.

Because the dataset lacked university, campus, and class identifiers, cluster-robust or multilevel models could not be estimated. Observed-score PROCESS analyses also do not account for measurement error in the multi-item constructs. These analytic constraints were acknowledged explicitly. All tests were two-sided, with *p* < 0.05 used as the statistical significance threshold; emphasis was placed on effect sizes and confidence intervals rather than statistical significance alone.

## 3 Results

### 3.1 Participant selection and sample characteristics

The exported dataset contained 2,255 responses. Sixteen records were excluded because age was outside 18–29 years, 80 postgraduate records were then excluded, and 420 remaining undergraduate records failed the prespecified attention check. The final analytic sample comprised 1,739 students. No item-level values were missing for the principal study variables. The sample included 889 men (51.1%) and 850 women (48.9%), with a mean age of 20.60 years (SD 1.56) (Table 1).

**Table 1:** Participant selection.

Stage	*n*	Excluded
Exported responses	2,255	–
Age outside 18–29 years	2,239	16
Postgraduate status	2,159	80
Failed attention check	1,739	420
Final analytic sample	1,739	–

### 3.2 Descriptive statistics and measurement diagnostics

The 41-item perceived campus walkability composite had a mean of 136.99 (SD 15.86; observed range 76–192). Exercise motivation averaged 58.00 (SD 11.68), general self-efficacy averaged 26.90 (SD 7.11), and the PARS-3 self-reported exercise behavior score averaged 16.81 (SD 19.88). The exercise score was right-skewed: its median was 8 (IQR 3–24), substantially below its mean. Accordingly, Spearman correlations and heteroscedasticity-consistent regression estimates were examined as sensitivity analyses (Tables 2, 3).

**Table 2:** Descriptive statistics of the principal variables.

Variable	Mean	SD	Median	IQR	Observed range
Walkability	136.99	15.86	137.0	127.0–147.5	76–192
Exercise motivation	58.00	11.68	59.0	49.0–67.0	15–75
General self-efficacy	26.90	7.11	26.0	21.0–30.0	10–40
Exercise behavior	16.81	19.88	8.0	3.0–24.0	0–100

**Table 3:** Descriptive statistics of the available campus-walkability domains.

Domain	Mean	SD	Observed range
Land-use mix / destination accessibility	43.43	9.93	0–65
Access to services	14.17	3.70	4–20
Street connectivity and pedestrian density	19.27	2.64	9–30
Pedestrian infrastructure and facilities	26.28	4.07	10–40
Aesthetics and environmental quality	18.33	3.30	5–25
Traffic safety	15.50	3.63	5–25

The peak-period pedestrian-density item listed in the questionnaire was absent from the analytic export; no value was imputed.

Cronbach’s alpha was 0.952 for the 15-item exercise-motivation composite and 0.949 for general self-efficacy. Motivation-domain alpha coefficients ranged from 0.766 to 0.833 (health = 0.822, weight/appearance = 0.790, enjoyment = 0.766, skill development = 0.790, and social interaction = 0.833). These coefficients indicate internal consistency but do not establish factorial or construct validity. Harman’s single-factor diagnostic identified no dominant single factor (first factor = 23.132%); however, common method variance cannot be excluded because all principal variables were measured by self-report at one time.

### 3.3 Distribution of self-reported exercise behavior

**Figure 1:**
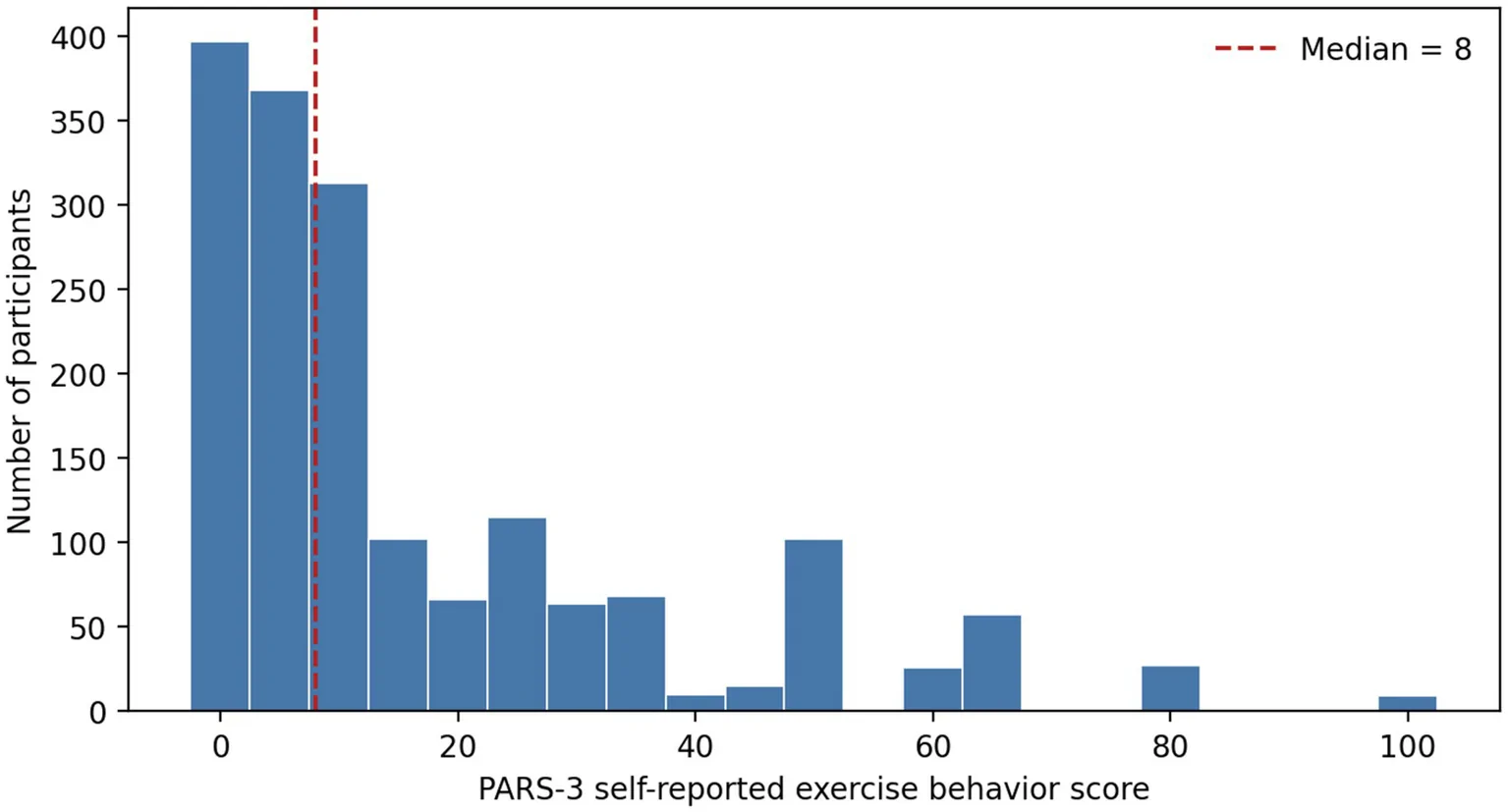
Distribution of the PARS-3 self-reported exercise behavior score.

For intensity, 714 students (41.1%) selected the light-exercise response category and 400 (23.0%) selected the low-intensity category. For duration, 522 (30.0%) reported 21–30 min per session. For frequency, 614 (35.3%) reported exercising 3–5 times per week. These labels reproduce the PARS-3 response options and should not be interpreted as objective intensity classifications (Figure 1 and Table 4).

**Table 4:** PARS-3 component distributions (*n* = 1,739).

Component	Response category	*n*	%
Intensity	Light exercise	714	41.1
	Low-intensity exercise	400	23.0
	Moderate-intensity exercise	264	15.2
	Vigorous, non-sustained exercise	303	17.4
	Vigorous, sustained exercise	58	3.3
Duration	<10 min	293	16.8
	11–20 min	450	25.9
	21–30 min	522	30.0
	31–59 min	217	12.5
	≥60 min	257	14.8
Frequency	<1 time/month	190	10.9
	2–3 times/month	305	17.5
	1–2 times/week	489	28.1
	3–5 times/week	614	35.3
	Approximately daily	141	8.1

### 3.4 Correlations among the principal variables

Pearson correlations were positive but small between perceived campus walkability and exercise behavior (*r* = 0.127), exercise motivation and exercise behavior (*r* = 0.142), and general self-efficacy and exercise behavior (*r* = 0.170); all *p* < 0.001. Walkability was correlated with exercise motivation (*r* = 0.237) and general self-efficacy (*r* = 0.215), while motivation and general self-efficacy were moderately correlated (*r* = 0.544). Spearman sensitivity estimates were similar in direction (*ρ* = 0.123–0.284 for associations involving walkability), indicating that the main correlation pattern was not dependent on Pearson assumptions (Table 5).

**Table 5:** Pearson correlations among the principal variables.

Variable	1	2	3	4
Walkability	–	–	–	–
Exercise motivation	0.237***	–	–	–
General self-efficacy	0.215***	0.544***	–	–
Exercise behavior	0.127***	0.142***	0.170***	–

1 = perceived campus walkability; 2 = exercise motivation; 3 = general self-efficacy; 4 = self-reported exercise behavior. ***p < 0.001.

### 3.5 Hierarchical regression of self-reported exercise behavior

The demographic and background model explained 20.7% of the variance in exercise behavior (adjusted *R*^2^ = 0.198). Adding perceived campus walkability increased *R*^2^ by 0.007 (Δ*F* = 14.34, *p* < 0.001). Adding exercise motivation and general self-efficacy produced a further Δ*R*^2^ of 0.013 (Δ*F* = 13.92, *p* < 0.001). The final model explained 22.6% of variance (adjusted *R*^2^ = 0.216); thus, the focal environmental and psychological variables added approximately 1.9 percentage points beyond the background model (Table 6).

**Table 6:** Focal coefficients in the fully adjusted hierarchical model.

Variable	*B*	HC3 SE	*β*	95% CI	*P*
Walkability	0.066	0.028	0.053	0.011 to 0.121	0.020
Exercise motivation	0.141	0.044	0.083	0.055 to 0.227	0.001
General self-efficacy	0.141	0.076	0.051	−0.008 to 0.291	0.064

Nominal covariates were dummy-coded. The model adjusted for sex, ethnicity, family residence, living expenses, grade, academic discipline, campus residence, perceived body shape, and family economic status.

Perceived campus walkability remained positively associated with exercise behavior (*B* = 0.066, 95% CI 0.011 to 0.121; standardized *β* = 0.053; *p* = 0.020). Exercise motivation was also positively associated with the outcome (*B* = 0.141, 95% CI 0.055 to 0.227; *β* = 0.083; *p* = 0.001). The coefficient for general self-efficacy was small and its HC3 confidence interval included zero (*B* = 0.141, 95% CI − 0.008 to 0.291; *β* = 0.051; *p* = 0.064). These estimates are associations and should not be interpreted as effects.

### 3.6 Cross-sectional serial indirect associations

Under the prespecified ordering of walkability → exercise motivation → general self-efficacy → exercise behavior, the total indirect association was 0.0369 (95% bootstrap CI 0.0208 to 0.0551). The indirect association through exercise motivation alone was 0.0242 (95% CI 0.0097 to 0.0411). In contrast, the interval included zero for the pathway through general self-efficacy alone (0.0051, 95% CI − 0.0004 to 0.0123) and for the serial pathway through motivation and general self-efficacy (0.0075, 95% CI − 0.0006 to 0.0158). The direct association was 0.0659 (95% CI 0.0110 to 0.1210), and the total association was 0.1028 (95% CI 0.0501 to 0.1571) (Table 7).

**Table 7:** Bootstrap indirect associations in prespecified and alternative models.

Model	Association	Effect	95% bootstrap CI
Prespecified	Via first mediator	0.0242	0.0097 to 0.0411
	Via second mediator	0.0051	−0.0004 to 0.0123
	Serial pathway	0.0075	−0.0006 to 0.0158
	Total indirect	0.0369	0.0208 to 0.0551
	Direct	0.0659	0.0110 to 0.1210
	Total	0.1028	0.0501 to 0.1571
Competing	Via first mediator	0.0126	−0.0011 to 0.0268
	Via second mediator	0.0134	0.0050 to 0.0241
	Serial pathway	0.0108	0.0044 to 0.0185
	Total indirect	0.0369	0.0208 to 0.0551
	Direct	0.0659	0.0110 to 0.1210
	Total	0.1028	0.0501 to 0.1571
Reverse direction	Via first mediator	0.0202	0.0108 to 0.0319
	Via second mediator	0.0042	0.0001 to 0.0097
	Serial pathway	0.0068	0.0030 to 0.0115
	Total indirect	0.0311	0.0191 to 0.0448
	Direct	0.0494	0.0078 to 0.0930
	Total	0.0805	0.0399 to 0.1241

Competing model: general self-efficacy before exercise motivation. Reverse-direction model: exercise behavior as the predictor and perceived walkability as the outcome. Confidence intervals are based on 5,000 bootstrap resamples. These cross-sectional models cannot identify temporal order or causality.

The competing ordering with general self-efficacy before exercise motivation also produced a nonzero serial indirect association (0.0108, 95% CI 0.0044 to 0.0185). The reverse-direction specification likewise produced nonzero indirect associations, including a serial association of 0.0068 (95% CI 0.0030 to 0.0115). Because several theoretically plausible orderings fit the cross-sectional associations, the data do not establish that motivation temporally precedes general self-efficacy or that perceived walkability initiates a causal mechanism.

### 3.7 Hypothesis evaluation

H1 was supported only as an adjusted cross-sectional association: more favorable perceived campus walkability was associated with higher self-reported exercise behavior, although the standardized coefficient was small. H2 was consistent with the data because the indirect association through exercise motivation excluded zero under the prespecified model. H3 was not supported because the bootstrap interval for the indirect association through general self-efficacy included zero. H4 was also not supported because the serial indirect-association interval included zero and competing and reverse-direction models produced plausible alternative patterns. The results therefore provide evidence of cross-sectional associations, not confirmation of a serial mediation mechanism.

## 4 Discussion

Among 1,739 undergraduate students, this study found small positive cross-sectional associations among perceived campus walkability, exercise motivation, general self-efficacy, and self-reported exercise behavior. After dummy coding categorical covariates and using HC3 robust standard errors, perceived campus walkability remained associated with exercise behavior (standardized *β* = 0.053), although its incremental contribution was modest. The indirect association through exercise motivation excluded zero, whereas the indirect association through general self-efficacy and the serial pathway did not. Competing and reverse-direction models also produced nonzero indirect associations. The findings therefore support cautious interpretation of correlated environmental perceptions and psychological characteristics, not a confirmed causal or temporally ordered mediation mechanism.

### 4.1 Perceived campus walkability and self-reported exercise behavior

More favorable perceived campus walkability was associated with higher PARS-3 exercise scores after adjustment for demographic and background characteristics. This direction is consistent with social ecological perspectives, which locate activity behavior within interacting individual, social, and environmental contexts (15). It also accords with evidence linking walkable environments to walking and daily steps (16, 17). However, the observed standardized coefficient was small, and adding walkability increased explained variance by only 0.7 percentage points. Statistical significance in this large sample should therefore not be equated with a large practical effect (18).

The exposure and outcome should also be distinguished carefully. The walkability measure assessed students’ perceptions of destination access, pedestrian conditions, environmental quality, and traffic safety, whereas PARS-3 primarily measured deliberate exercise intensity, duration, and frequency. It did not comprehensively measure campus walking, active commuting, daily steps, or total movement. Campus walkability may be more directly related to those unmeasured behaviors than to deliberate exercise. Accordingly, the present association is informative but does not demonstrate that a walkable campus increases total physical activity.

Perceived walkability is not equivalent to objective campus design. Students who use more campus spaces or exercise more frequently may become more familiar with destinations and may rate the environment more favorably. Conversely, negative affect, low perceived fitness, or limited campus use could influence both environmental evaluations and exercise reports. Future studies should combine perceived measures with GIS indicators, field audits, route-level observations, accelerometry, step counts, and GPS data to separate subjective experience from objective environmental exposure.

### 4.2 Exercise motivation as an indirect correlate

The indirect association through exercise motivation was statistically distinguishable from zero under the prespecified model. A campus perceived as accessible, safe, and pleasant may coincide with fewer perceived barriers and more favorable reasons for exercise. Nevertheless, the motivation instrument assessed health, appearance, enjoyment, skill, and social motives rather than autonomous and controlled regulations. The findings should therefore not be presented as a direct test of self-determination theory or as evidence that campus walkability causes autonomous motivation (10).

Exercise motivation showed the largest focal standardized coefficient in the fully adjusted outcome model (*β* = 0.083), but its magnitude remained small. Practical strategies may combine accessible walking routes with enjoyable low-threshold activities, peer opportunities, and skill-development programs. Such strategies are plausible applications rather than effects demonstrated by this cross-sectional study.

### 4.3 General self-efficacy and uncertain temporal ordering

The analysis did not provide clear evidence of an indirect association through general self-efficacy alone because the 95% bootstrap confidence interval included zero. General self-efficacy also had a small outcome-model coefficient whose HC3 confidence interval included zero. Therefore, the findings did not support an independent indirect association through general self-efficacy.

The measure assessed broad confidence in coping with difficult or unexpected situations, not confidence in exercising despite fatigue, time pressure, weather, low mood, or other activity-specific barriers. It is therefore inappropriate to equate this construct with exercise confidence. General self-efficacy may be a relatively stable personal characteristic, may precede motivation, or may be strengthened by successful exercise experiences. Exercise-specific or barrier-specific self-efficacy measures would be better aligned with a proposed exercise mechanism.

### 4.4 Serial indirect associations and competing models

The prespecified serial indirect association from walkability through exercise motivation and then general self-efficacy had a bootstrap confidence interval that included zero. H4 was therefore not supported. Moreover, a competing model that placed general self-efficacy before exercise motivation produced a nonzero serial association, and a reverse-direction model beginning with exercise behavior also produced nonzero indirect associations. These results demonstrate that statistically estimable indirect pathways are compatible with several orderings of the same cross-sectional variables.

PROCESS Model 6 decomposes regression associations under an assumed order; it does not determine temporal precedence or verify a psychological mechanism. Students who exercise more may report stronger motivation and efficacy and may evaluate campus spaces more favorably because they use them more often. Longitudinal studies with at least three measurement occasions, natural experiments following campus modifications, or cross-lagged and multilevel designs would be needed to evaluate directionality more appropriately.

### 4.5 Implications for university health promotion

The results support a proportionate, multicomponent interpretation. Universities may assess route continuity, lighting, pedestrian–vehicle separation, wayfinding, destination accessibility, greenery, and rest facilities while also offering enjoyable and socially supportive opportunities for exercise (19–23). Because the focal variables added only about 1.9 percentage points of explained variance beyond background characteristics, environmental and psychological initiatives should complement rather than replace physical education, sports services, workload management, social support, and attention to socioeconomic barriers.

The dimension-level sensitivity analysis identified destination accessibility as the only walkability domain with a confidence interval excluding zero when all six available domains were entered together. This exploratory result may help prioritize convenient connections among dormitories, teaching buildings, dining facilities, libraries, and sports destinations (24). However, domain findings require replication because the campus-adapted scale was not fully validated and one questionnaire item was absent from the analytic export.

### 4.6 Strengths and limitations

Strengths include the large analytic sample, explicit dummy coding of categorical covariates, hierarchical regression, heteroscedasticity-consistent HC3 standard errors, bootstrap confidence intervals, and comparison of alternative cross-sectional orderings. The study also distinguishes perceived walkability from objective design, deliberate exercise from total physical activity, and general from exercise-specific self-efficacy.

Several limitations are substantial. First, the cross-sectional design precludes temporal and causal inference and is vulnerable to reverse causality and residual confounding (18). Second, all principal variables were self-reported at one time; Harman’s diagnostic cannot exclude common method variance, recall error, social desirability, or favorable-response tendencies. Third, PARS-3 does not comprehensively capture campus walking, active commuting, or total movement and was not validated against an objective criterion in this sample. Fourth, the general self-efficacy measure did not assess exercise-specific confidence.

Fifth, the campus walkability questionnaire was adapted from NEWS without a fully documented translation and validation process. The questionnaire listed 42 substantive items, but the analytic dataset contained 41; the peak-period pedestrian-density item was absent and was not imputed. Different response formats were combined, and the structural validity of the total score remains uncertain. Sixth, the motivation total combined conceptually different motive domains, although domain-specific reliability was reported. Seventh, participants were recruited through nonprobability convenience sampling from six undergraduate universities across Shandong Province. Volunteer-assisted recruitment may have introduced self-selection bias, thereby limiting the representativeness and generalizability of the findings to the broader university student population. Although the online survey platform required participants to log in and restricted submissions to one per authenticated account, device, and IP address, these identifiers were not retained in the de-identified analytic dataset. Consequently, the effectiveness of these duplicate-submission controls could not be independently verified. Eighth, individual-level university, campus, and class identifiers were not retained, precluding cluster-robust or multilevel analyses. The HC3 standard errors used in this study address heteroscedasticity but do not account for university-, campus-, or class-level clustering. If responses were correlated within universities, campuses, or classes, the standard errors may have been underestimated and the confidence intervals may be overly narrow. Finally, arts and sports-related majors were combined in the source category, so confounding by compulsory sports training could not be isolated. These limitations restrict the generalizability and statistical precision of the findings and require cautious interpretation.

## 5 Conclusion

Perceived campus walkability was weakly associated with self-reported exercise behavior among 1,739 undergraduate students after adjustment for demographic and background characteristics. Exercise motivation showed a nonzero indirect association under the prespecified cross-sectional model, whereas indirect associations through general self-efficacy alone and through the proposed serial pathway were not statistically distinguishable from zero. Competing and reverse-direction models were also plausible. The findings do not confirm a causal mediation mechanism or temporal sequence.

Universities may consider environmental accessibility and supportive exercise opportunities as complementary components of health promotion, while recognizing that the observed associations were small and that PARS-3 did not measure total campus walking. Longitudinal, multilevel, and natural-experimental studies using validated campus-environment instruments, exercise-specific efficacy measures, and objective activity data are needed before effects of campus modifications can be inferred.

## Data Availability

The raw data supporting the conclusions of this article will be made available by the authors, without undue reservation.
