# Supplementary material for: Associations between perceived campus walkability and self-reported exercise behavior among college students: a cross-sectional serial indirect-effects study of exercise motivation and general self-efficacy
Source: Front Public Health. 2026 Sep 4;14:1871624. doi: 10.3389/fpubh.2026.1871624 (PMC13585724; doi:10.3389/fpubh.2026.1871624)
Supplement: Supplementary file 1 [file Data_Sheet_1.docx]

**Supplementary Materials**

The supplementary materials comprise the following tables, note, and appendices. All newly added material is shown in red.

| No. | Supplementary item |
| --- | --- |
| 1 | Supplementary Table S1. Full Multivariable Regression Results and Multicollinearity Diagnostics |
| 2 | Supplementary Table S2. Spearman Correlations Among the Main Study Variables |
| 3 | Supplementary Table S3. Sensitivity Analyses of Campus Walkability Domains and Self-Reported Exercise Behavior |
| 4 | Supplementary Table S4. Alternative Cross-Sectional Indirect-Effects Models |
| 5 | Supplementary Note S1. Data Cleaning, Variable Scoring, and Participant Selection |
| 6 | Appendix 1. Physical Activity Rating Scale-3 (PARS-3) |
| 7 | Appendix 2. Campus-Adapted Perceived Walkability Questionnaire |
| 8 | Appendix 3. Abbreviated Exercise Motivation Questionnaire |
| 9 | Appendix 4. General Self-Efficacy Scale |
| 10 | Appendix 5. Scoring and Analytic Coding Summary |

**Supplementary Table S1. Full Multivariable Regression Results and Multicollinearity Diagnostics**

| Term | B | HC3 SE | 95% CI | P | VIF |
| --- | --- | --- | --- | --- | --- |
| Intercept | -18.757 | 9.358 | -37.110 to -0.403 | .045 | — |
| Age | -0.027 | 0.392 | -0.796 to 0.743 | .945 | 1.85 |
| Sex: male | 13.961 | 0.939 | 12.120 to 15.803 | <.001 | 1.16 |
| Ethnicity: minority | -0.087 | 0.878 | -1.810 to 1.636 | .921 | 1.04 |
| Family residence: town | -0.264 | 1.384 | -2.978 to 2.450 | .849 | 1.65 |
| Family residence: rural | 0.400 | 1.218 | -1.989 to 2.788 | .743 | 1.85 |
| Monthly expenses: CNY 1000–2000 | 2.038 | 0.990 | 0.097 to 3.979 | .040 | 1.35 |
| Monthly expenses: >CNY 3000 | 8.565 | 4.743 | -0.738 to 17.868 | .071 | 1.21 |
| Monthly expenses: CNY 2000–3000 | 3.232 | 2.371 | -1.418 to 7.883 | .173 | 1.32 |
| Grade: fourth year | 4.493 | 1.984 | 0.600 to 8.385 | .024 | 1.74 |
| Grade: third year | 1.062 | 1.464 | -1.810 to 3.934 | .468 | 1.76 |
| Grade: second year | 1.543 | 1.136 | -0.685 to 3.771 | .175 | 1.26 |
| Discipline: engineering | -0.002 | 1.219 | -2.393 to 2.390 | .999 | 1.58 |
| Discipline: science | 1.158 | 0.960 | -0.726 to 3.041 | .228 | 1.62 |
| Discipline: arts/sports | 10.764 | 1.415 | 7.988 to 13.540 | <.001 | 1.56 |
| Campus residence: yes | 3.773 | 2.918 | -1.952 to 9.497 | .196 | 1.03 |
| Body shape: underweight | -1.731 | 1.213 | -4.110 to 0.647 | .154 | 1.09 |
| Body shape: overweight | -2.307 | 1.059 | -4.384 to -0.231 | .029 | 1.08 |
| Body shape: obese | -6.257 | 2.231 | -10.633 to -1.881 | .005 | 1.05 |
| Family economy: difficult | -0.836 | 0.924 | -2.648 to 0.976 | .365 | 1.17 |
| Family economy: affluent | -1.092 | 4.071 | -9.077 to 6.893 | .789 | 1.13 |
| Perceived campus walkability | 0.066 | 0.028 | 0.011 to 0.121 | .019 | 1.09 |
| Exercise motivation | 0.141 | 0.044 | 0.055 to 0.227 | .001 | 1.48 |
| General self-efficacy | 0.141 | 0.076 | -0.009 to 0.291 | .065 | 1.51 |

Note. Outcome: PARS-3 self-reported exercise behavior score. Reference categories were female sex, Han ethnicity, urban family residence, monthly expenses <CNY 1000, first-year grade, humanities/social sciences, non-campus residence, normal perceived body shape, and average family economic status. All VIF values were below 2.0.

**Supplementary Table S2. Spearman Correlations Among the Main Study Variables**

| Variable | 1 | 2 | 3 | 4 |
| --- | --- | --- | --- | --- |
| Walkability | — | — | — | — |
| Exercise motivation | 0.284*** | — | — | — |
| General self-efficacy | 0.220*** | 0.507*** | — | — |
| Exercise behavior | 0.123*** | 0.170*** | 0.186*** | — |

Note. 1=perceived campus walkability; 2=exercise motivation; 3=general self-efficacy; 4=self-reported exercise behavior. ***P<.001.

**Supplementary Table S3. Sensitivity Analyses of Campus Walkability Domains and Self-Reported Exercise Behavior**

| Domain | B | HC3 SE | 95% CI | P |
| --- | --- | --- | --- | --- |
| Land-use mix / destinations | 0.128 | 0.046 | 0.037 to 0.218 | .006 |
| Access to services | 0.050 | 0.175 | -0.293 to 0.392 | .776 |
| Street connectivity / pedestrian density | 0.098 | 0.230 | -0.353 to 0.549 | .670 |
| Pedestrian infrastructure / facilities | -0.041 | 0.166 | -0.365 to 0.284 | .807 |
| Aesthetics / environmental quality | 0.251 | 0.191 | -0.123 to 0.625 | .189 |
| Traffic safety | 0.155 | 0.134 | -0.108 to 0.417 | .249 |

Note. The six domains were entered simultaneously with the demographic and background covariates. The analysis is exploratory because the campus-adapted domain structure was not fully validated.

**Supplementary Table S4. Alternative Cross-Sectional Indirect-Effects Models**

| Model | Association | Effect | 95% bootstrap CI |
| --- | --- | --- | --- |
| Prespecified: motivation → efficacy | Via first mediator | 0.0243 | 0.0096 to 0.0413 |
|  | Via second mediator | 0.0051 | -0.0004 to 0.0123 |
|  | Serial pathway | 0.0076 | -0.0007 to 0.0159 |
|  | Total indirect | 0.0370 | 0.0209 to 0.0554 |
| Competing: efficacy → motivation | Via first mediator | 0.0127 | -0.0006 to 0.0275 |
|  | Via second mediator | 0.0135 | 0.0050 to 0.0245 |
|  | Serial pathway | 0.0108 | 0.0042 to 0.0184 |
|  | Total indirect | 0.0370 | 0.0214 to 0.0555 |
| Reverse direction | Via first mediator | 0.0204 | 0.0107 to 0.0323 |
|  | Via second mediator | 0.0041 | 0.0002 to 0.0096 |
|  | Serial pathway | 0.0067 | 0.0029 to 0.0112 |
|  | Total indirect | 0.0312 | 0.0190 to 0.0448 |

Note. Confidence intervals were based on 5,000 bootstrap resamples. These models decompose cross-sectional associations under assumed orderings and do not identify temporal precedence or causality.

**Supplementary Note S1. Data Cleaning, Variable Scoring, and Participant Selection**

The raw survey export contained 2,255 records. Sixteen records outside the prespecified age range of 18–29 years were excluded, followed by 80 postgraduate records and 420 remaining undergraduate records that failed the embedded attention check. The final analytic sample comprised 1,739 participants. The questionnaire listed 42 substantive campus-walkability items, whereas the export contained 41; the peak-period pedestrian-density item was absent and was not imputed. The 13 destination responses were scored from 5 (1–5 minutes) to 1 (>30 minutes), and “do not know” was coded 0. The 28 available agreement items were scored 1–5, with negatively worded items reverse-coded. PARS-3 was calculated as intensity (1–5) × duration (0–4) × frequency (1–5).

**Appendix 1. Physical Activity Rating Scale-3 (PARS-3)**

English translation of the Chinese-language items administered in the survey. Participants selected one response for each item.

| Item | Component | Response category | Score |
| --- | --- | --- | --- |
| 1 | Exercise intensity | Light exercise (eg, walking or radio calisthenics) | 1 |
| 1 |  | Low-intensity, nonstrenuous exercise (eg, recreational volleyball, table tennis, jogging, or tai chi) | 2 |
| 1 |  | Moderate-intensity, relatively vigorous and sustained exercise (eg, cycling or running) | 3 |
| 1 |  | Vigorous but nonsustained exercise causing rapid breathing and heavy sweating (eg, badminton, basketball, tennis, or football) | 4 |
| 1 |  | Vigorous and sustained exercise causing rapid breathing and heavy sweating (eg, racing, aerobics, or swimming) | 5 |
| 2 | Duration per session | <10 minutes | 0 |
| 2 |  | 11–20 minutes | 1 |
| 2 |  | 21–30 minutes | 2 |
| 2 |  | 31–59 minutes | 3 |
| 2 |  | ≥60 minutes | 4 |
| 3 | Frequency | <1 time/month | 1 |
| 3 |  | 2–3 times/month | 2 |
| 3 |  | 1–2 times/week | 3 |
| 3 |  | 3–5 times/week | 4 |
| 3 |  | Approximately daily | 5 |

Total score=intensity × duration × frequency; theoretical range=0–100.

**Appendix 2. Campus-Adapted Perceived Walkability Questionnaire**

English translation of the Chinese-language questionnaire. Items 1–13 used walking-time response categories; Items 14–42 used a 5-point agreement scale. Item 20 appeared in the questionnaire but was absent from the analytic export.

| Item | Item wording | Response options | Analytic direction |
| --- | --- | --- | --- |
| 1 | Walking time from the dormitory to the Teaching building. | 1–5, 6–10, 11–20, 20–30, >30 minutes; do not know | Direct (shorter time=higher score) |
| 2 | Walking time from the dormitory to the Library. | 1–5, 6–10, 11–20, 20–30, >30 minutes; do not know | Direct (shorter time=higher score) |
| 3 | Walking time from the dormitory to the Dining hall. | 1–5, 6–10, 11–20, 20–30, >30 minutes; do not know | Direct (shorter time=higher score) |
| 4 | Walking time from the dormitory to the Fast-food restaurant. | 1–5, 6–10, 11–20, 20–30, >30 minutes; do not know | Direct (shorter time=higher score) |
| 5 | Walking time from the dormitory to the Fruit shop. | 1–5, 6–10, 11–20, 20–30, >30 minutes; do not know | Direct (shorter time=higher score) |
| 6 | Walking time from the dormitory to the Parcel station. | 1–5, 6–10, 11–20, 20–30, >30 minutes; do not know | Direct (shorter time=higher score) |
| 7 | Walking time from the dormitory to the Supermarket. | 1–5, 6–10, 11–20, 20–30, >30 minutes; do not know | Direct (shorter time=higher score) |
| 8 | Walking time from the dormitory to the Department store. | 1–5, 6–10, 11–20, 20–30, >30 minutes; do not know | Direct (shorter time=higher score) |
| 9 | Walking time from the dormitory to the Gymnasium. | 1–5, 6–10, 11–20, 20–30, >30 minutes; do not know | Direct (shorter time=higher score) |
| 10 | Walking time from the dormitory to the Outdoor sports facility. | 1–5, 6–10, 11–20, 20–30, >30 minutes; do not know | Direct (shorter time=higher score) |
| 11 | Walking time from the dormitory to the Hospital. | 1–5, 6–10, 11–20, 20–30, >30 minutes; do not know | Direct (shorter time=higher score) |
| 12 | Walking time from the dormitory to the Campus shuttle stop. | 1–5, 6–10, 11–20, 20–30, >30 minutes; do not know | Direct (shorter time=higher score) |
| 13 | Walking time from the dormitory to the Green space. | 1–5, 6–10, 11–20, 20–30, >30 minutes; do not know | Direct (shorter time=higher score) |
| 14 | Teaching buildings are within a short walking distance of my dormitory. | Strongly disagree to strongly agree | Direct |
| 15 | Shops are within a short walking distance of my dormitory. | Strongly disagree to strongly agree | Direct |
| 16 | Commercial areas are easy to reach by public transportation. | Strongly disagree to strongly agree | Direct |
| 17 | Many destinations can be reached on foot from my dormitory. | Strongly disagree to strongly agree | Direct |
| 18 | Many slopes make walking difficult. | Strongly disagree to strongly agree | Reverse |
| 19 | Many obstacles make walking difficult. | Strongly disagree to strongly agree | Reverse |
| 20 | There are many pedestrians during peak periods. | Strongly disagree to strongly agree | Not scored (absent from export) |
| 21 | There are many pedestrians during nonpeak periods. | Strongly disagree to strongly agree | Direct |
| 22 | Campus streets have few or no dead ends. | Strongly disagree to strongly agree | Direct |
| 23 | Distances between intersections on campus are usually short. | Strongly disagree to strongly agree | Direct |
| 24 | There are many different routes from one place to another. | Strongly disagree to strongly agree | Direct |
| 25 | Most campus streets have sidewalks. | Strongly disagree to strongly agree | Direct |
| 26 | Campus sidewalks are crowded with parked cars. | Strongly disagree to strongly agree | Reverse |
| 27 | There is a separation between the roadway and sidewalk on campus. | Strongly disagree to strongly agree | Direct |
| 28 | Streets are well lit at night. | Strongly disagree to strongly agree | Direct |
| 29 | Busy streets have pedestrian crossings and traffic signals. | Strongly disagree to strongly agree | Direct |
| 30 | There are indoor spaces where people can walk on campus. | Strongly disagree to strongly agree | Direct |
| 31 | Campus streets and sidewalks are usually wet. | Strongly disagree to strongly agree | Reverse |
| 32 | Rest facilities such as benches are available. | Strongly disagree to strongly agree | Direct |
| 33 | There are trees along campus streets. | Strongly disagree to strongly agree | Direct |
| 34 | There are many interesting things to see while walking on campus. | Strongly disagree to strongly agree | Direct |
| 35 | There are many green spaces on campus. | Strongly disagree to strongly agree | Direct |
| 36 | There are attractive buildings on campus. | Strongly disagree to strongly agree | Direct |
| 37 | Air pollution on campus is usually high. | Strongly disagree to strongly agree | Reverse |
| 38 | Traffic on nearby streets is very congested and makes walking difficult. | Strongly disagree to strongly agree | Reverse |
| 39 | Traffic speeds on most nearby streets are usually slow (≤30 km/h). | Strongly disagree to strongly agree | Direct |
| 40 | Most drivers exceed the speed limit on campus. | Strongly disagree to strongly agree | Reverse |
| 41 | Many cars are parked on nearby streets, making it difficult to cross. | Strongly disagree to strongly agree | Reverse |
| 42 | Many vehicles pass on nearby streets, making road crossing frightening. | Strongly disagree to strongly agree | Reverse |

**Appendix 3. Abbreviated Exercise Motivation Questionnaire**

| Item | Item wording | Domain |
| --- | --- | --- |
| 1 | I want to have a strong body. | Health |
| 2 | I want to control my weight. | Weight/appearance |
| 3 | I want to participate in enjoyable recreational activities. | Enjoyment |
| 4 | I want to acquire new exercise skills. | Skill development |
| 5 | I want to meet new friends. | Social interaction |
| 6 | I want to maintain physical and mental health. | Health |
| 7 | I want to maintain or improve my body shape. | Weight/appearance |
| 8 | I want to maintain a pleasant mood. | Enjoyment |
| 9 | I want to improve my current exercise skills. | Skill development |
| 10 | I want to strengthen affection and friendship with friends. | Social interaction |
| 11 | I want to live a healthy life. | Health |
| 12 | I want to make my appearance more attractive. | Weight/appearance |
| 13 | I want to enjoy a happy life. | Enjoyment |
| 14 | I want to maintain my current level of exercise skills. | Skill development |
| 15 | I want to maintain good social relationships. | Social interaction |

Response options: 1=completely inconsistent, 2=inconsistent, 3=generally consistent, 4=consistent, and 5=completely consistent. No item was reverse-scored.

**Appendix 4. General Self-Efficacy Scale**

| Item | Item wording |
| --- | --- |
| 1 | If I try hard enough, I can always manage to solve difficult problems. |
| 2 | If someone opposes me, I can find the means and ways to get what I want. |
| 3 | It is easy for me to stick to my aims and accomplish my goals. |
| 4 | I am confident that I could deal efficiently with unexpected events. |
| 5 | Thanks to my resourcefulness, I know how to handle unforeseen situations. |
| 6 | I can solve most problems if I invest the necessary effort. |
| 7 | I can remain calm when facing difficulties because I can rely on my coping abilities. |
| 8 | When I am confronted with a problem, I can usually find several solutions. |
| 9 | If I am in trouble, I can usually think of a solution. |
| 10 | I can usually handle whatever comes my way. |

Response options: 1=not at all true, 2=hardly true, 3=moderately true, and 4=exactly true. No item was reverse-scored.

**Appendix 5. Scoring and Analytic Coding Summary**

**Perceived campus walkability.** Thirteen destination walking-time items were coded 5, 4, 3, 2, and 1 from the shortest to longest time category; “do not know” was coded 0. The 28 available agreement items were coded 1–5, and negatively worded items were reversed as 6−response. The peak-period pedestrian-density item appearing in the questionnaire was absent from the analytic export and was not scored.

**PARS-3.** Intensity=1–5; duration=0–4; frequency=1–5. Total=intensity×duration×frequency (theoretical range 0–100). The outcome represents self-reported deliberate exercise behavior rather than total physical activity.

**Exercise motivation.** Fifteen items, five domains of three items each, scored 1–5 with no reverse items. Domain range=3–15; total range=15–75.

**General self-efficacy.** Ten items scored 1–4 with no reverse items. Total=sum of the 10 items (range 10–40).

**Categorical covariates.** Sex and other binary variables were entered using one indicator. Grade, academic discipline, family residence, living-expense category, perceived body shape, and family economic status were represented by k−1 indicators, with the reference category stated in each analysis. Arts and sports-related students could not be separated because they were combined in the source response category.
